# Fluorine-Rich Planetary Environments as Possible Habitats for Life 

**DOI:** 10.3390/life4030374

**Published:** 2014-08-18

**Authors:** Nediljko Budisa, Vladimir Kubyshkin, Dirk Schulze-Makuch

**Affiliations:** 1 Department of Chemistry, Technical University of Berlin, Müller-Breslau-Straße 10, 10623 Berlin, Germany; E-Mail: kubyshkin@win.tu-berlin.de; 2 School of the Environment, Washington State University, Webster Hall 1148, Pullman, WA 99164, USA; E-Mail: dirksm@wsu.edu; 3 Center for Astronomy and Astrophysics, Technical University of Berlin, Hardenbergstraße 36, 10623 Berlin, Germany

**Keywords:** fluorine, habitability, life, planetary body, atmosphere, solvent, organic synthesis, organic chemistry, alternative biochemistry

## Abstract

In polar aprotic organic solvents, fluorine might be an element of choice for life that uses selected fluorinated building blocks as monomers of choice for self-assembling of its catalytic polymers. Organofluorine compounds are extremely rare in the chemistry of life as we know it. Biomolecules, when fluorinated such as peptides or proteins, exhibit a “*fluorous* effect”,* i.e.*, they are fluorophilic (neither hydrophilic nor lipophilic). Such polymers, capable of creating self-sorting assemblies, resist denaturation by organic solvents by exclusion of fluorocarbon side chains from the organic phase. *Fluorous* cores consist of a compact interior, which is shielded from the surrounding solvent. Thus, we can anticipate that fluorine-containing “teflon”-like or “non-sticking” building blocks might be monomers of choice for the synthesis of organized polymeric structures in fluorine-rich planetary environments. Although no fluorine-rich planetary environment is known, theoretical considerations might help us to define chemistries that might support life in such environments. For example, one scenario is that all molecular oxygen may be used up by oxidation reactions on a planetary surface and fluorine gas could be released from F-rich magma later in the history of a planetary body to result in a fluorine-rich planetary environment.

## 1. Introduction

Fluorine is the 24th most abundant element in the universe (4 × 10^−5^%), and thus relatively rare. However, in Earth’s crust it is enriched and is the 13th most abundant element by weight percent (0.054%), just ahead of carbon (0.02%). The elements to which fluorine has a high affinity, Si, Al, Ca, and Mg are also common, both in Earth’s crust and the universe (28.2/0.07%, 8.1/0.005%, 4.1/0.007%, and 2.3/0.06%, respectively). This is why fluorine is likely to be found tightly bound within stable substances outside of liquid conditions.

Chemically fluorine is the most electronegative element in the periodic table and can be considered as an alternative substitute to oxygen. However, oxygen is much more abundant: five orders of magnitude more common in the universe and three orders of magnitude more common in Earth’s crust. Oxygen’s dominance compared to fluorine becomes even more apparent when comparing the abundance of these elements in organisms on Earth: hydrogen and oxygen are the most common elements [1,2], while fluorine is only a rare trace element, which does not appear to be essential to life. In Earth’s crust, fluorine occurs most commonly as calcium fluoride being a main constituent of minerals such as fluoroapatite, cryolyte and topaz.

Although fluorine abundance in Earth’s crust is quite high, it is rare in the oceans, probably due to the low water solubility of its salts (fluorides). This is why, for instance, chlorides are much more common in sea water than fluorides (19,000* vs.* 1.3 ppm) despite the latter being more abundant in Earth’s crust [3,4]. This might also be one reason for the rarity of metabolically generated fluorinated compounds (only 30 naturally occurring organofluorine compounds are found so far and one enzyme—fluorinase—that catalyzes C–F bond formation), whereas there exist more than 3500 organohalogens of biogenic origin [4,5]. All halogens, including fluorine, initially arrive at the planetary surface through volcanic activity, particularly geothermal processes [6].

While biogenic fluorine compounds are quite rare, there is a large number and range of commercially generated fluorinated compounds, which are all of anthropogenic origin [7]. Thus, even though fluorine is rarely utilized by life as we know it, it does have great application potential for biotechnical purposes. Of special interest in this context are attempts to generate biochemical structures with fluorine—mostly polymers of amino acids (proteins) and nucleic acids (DNA/RNA), but also lipids and small metabolites and metabolic intermediates (4-fluorothreonine, fluoroacetate, fluorocitrate *etc.*; for more information see [8]). Life appears not to have used fluoroorganic motifs due to the low availability of fluorides in solution and its relative low availability compared to direct competitors of fluorine such as oxygen, chlorine, and hydrogen.

## 2. Hydrofluoric Acid as a Solvent for Life?

Water is certainly a solvent of enormous importance for the evolution of life on Earth. However, there are alternative solvents and hydrofluoric acid (HF) is one of these with a higher potential to replace water [9]. Hydrofluoric acid has some similar properties to water. Its total liquid range at a pressure of 1 bar is 103.4 degrees, extending from −83.4 °C to +20.0 °C. Thus, HF as a solvent is available at lower temperatures than water, thus being more suitable for somewhat colder planetary bodies. Its enthalpy of fusion and enthalpy of vaporization are 4.6 and 30.3 kJ/mol, respectively, just slightly lower than water, which means that it is still a good moderator of temperature extremes. HF’s dielectric constant is slightly larger (83.6* vs.* 80.1), and its dipole moment is slightly lower than for water (1.83* vs.* 1.85). However, the viscosity of hydrofluoric acid is only about half of that of water and its surface tension is 4 times smaller (18.1 × 10^−3^ J/m^2^* vs.* 71.99 × 10^−3^ J/m^2^). Both water and HF are hydrogen bond forming, autoprotolytic solvents. In addition the diffusion of charges can proceed through the hydrogen bond network (structural diffusion) in both media. Therefore HF is able to maintain the acid-base chemistry in the same way as water.

One particular property, which is attributed to water and can be considered as essential for life is the existence of the hydrocarbon phase segregation due to the hydrophobic effect. Interestingly, non-polar hydrocarbon compounds are only poorly soluble in hydrofluoric acid, but many of them are polymerized, decomposed or lead to conducting solutions with complex cations, in which the organic molecule bonds to the proton of HF [10]. Thus, if HF would occur in a planetary environment in sufficient quantity it could in principle be a suitable solvent for organic compounds, either by itself or in an aqueous solution.

Pure HF is one of the strongest acids found and has a universal acidity parameter H_0_ of −15.0 [11]. Thus, it would be extremely unlikely to be used by biology. Solvation of HF in water extends it to a medium strong acid (pK_a_ of 3.19 [12]), which makes it more suitable to be used by life as a solvent. Nevertheless, a planet on which a HF-H_2_O mixture would exist as the sole potential solvent would certainly require the development of a different biochemistry, such as a different set of macromolecules that would take the function of nucleic acids (as informational polymers) and proteins (as catalytic polymers) than used by life as we know it. Liquid HF would also arrest Ca^2+^ and Mg^2+^ ions in liquid medium and exclude their function as adhesive bridges, skeletal elements, secondary messengers* etc.* Therefore, it would be necessary to develop another inorganic biochemistry for the hypothetical metabolism wherein HF is used as the solvent.

## 3. Fluorine-Containing Organic Compounds as Possible Building Blocks of Life

### 3.1. Fluorine-Containing Organic Compounds

Fluorine is largely ignored by the biosphere as a building block for life. But humans have been profiting from organofluorine chemistry for a long time. The remarkable development of organofluorine chemistry in material and medicinal science has proliferated due to the properties of fluorine on the molecular level. A good example is 5-fluorouracil, which is used as a common anticancer drug for more than 50 years due to similarity of this molecule with the natural nucleobase uracil [13]. The cell machinery would take fluorouracil as a substrate for thymidine production because the C–H (in uracil) and C–F (in fluorouracil) groups are so similar in size. However, the stability and polarity inversion in the latter case would inhibit essential methylation on this group, e.g., blocking the DNA synthesis. This example represents the medicinal chemistry approach of site-specific fluorine modifications in nature-like substrate molecules [14]. Fluorine can serve for a somewhat biocompatible tuning of the molecular properties such as polarity, sterical size, lipo- and hydrophilicity [15]. The presence of fluorine atoms in an organic chemical species increases the polarity of that substance. Conversely, hydrophobicity of the substance can be increased by addition of polyfluorinated groups in the case of CF_3_–, SF_5_– or similar moieties. Further increased levels of fluorination in an aliphatic chain leads then to the appearance of the *fluorous* effect (*vide infra*), which cannot be explicitly described in the terms of hydrophilic-lipophilic dualism, opening an entirely new opportunity for phase segregation.

The electron withdrawing effect of fluorine in aromatic structures such as the pentafluorophenyl-group significantly enhances the strength of π-stacking interaction [16,17]. In particular it was demonstrated that perfluorinated aromatic rings can serve for a DNA double strand stability based on the hydrophobicity motif as an alternative to Watson-Crick paring [18].

### 3.2. Fluorous Effect and Folding of Highly Fluorinated Bio-Molecules

**Figure 1:**
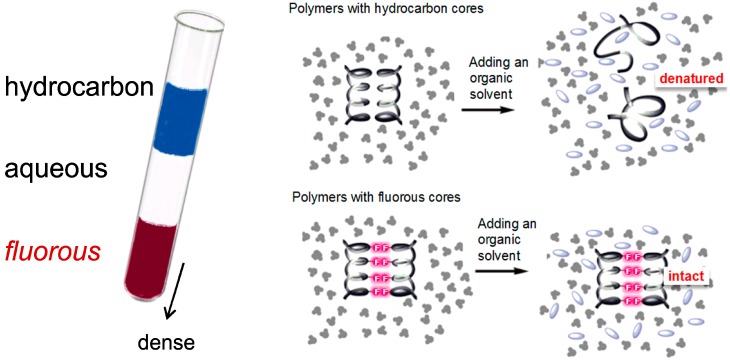
Physiochemical nature of *fluorous* effect. **Left**: phase separation of perfluorinated compounds/solvents which are immiscible with both organic and aqueous phases. In the contemporary literature, the term “*fluorous*” is used for highly fluorinated (*i.e.*, perfluorinated) solvents, in an analogy “aqueous” for water-based systems [30]. **Right**: hydrocarbon* versus* *fluorous* protein core (dark structures—water solvent; faint spots—organic solvent). The hydrocarbon core of a polymer (Top) opens up upon addition of an organic solvent leading to denaturation, whereas a *fluorous* core (Bottom) remains intact. The *fluorous* effect is very similar to the effect responsible for the non-sticking properties of Teflon [31]. In general, polymeric materials modified by multiple fluorinated carbons possess elevated hydrophobicity and stability. These considerations might be of particular importance in assessing whether life might be able to exist in hydrophobic solvents [9,32] under “reverse phase” conditions. Figure modified from [33].

Increased fluorine content in an aliphatic organic molecule or polymer leads to the separation of the highly-fluorinated species into a special phase, which is neither hydrophilic nor lipophilic in their standard meanings. The extreme would be Teflon^®^, the most chemically and solvent-inert organic polymer. In addition, the high density of perfluorinated compounds causes the phase segregation in a macroscopic manner (Figure 1). Highly fluorinated substances are called *fluorous* and their properties have found numerous applications in the context of material science [19]. The perfluorinated chains were found to form helices and double helices producing highly ordered liquid crystals within the hydrophobic environments [20]. This property is of high importance considering ordered self-assembly of biological molecules and molecular machines.

Classical *fluorous* materials are composed of perfluorinated F_2n+1_C_n_–chains followed by a “normal” hydrocarbon spacer and the functional group. These constructs called *ponytails* were used as building blocks for phospholipids. Interestingly, it was demonstrated that such lipids are prone to build lamellar phases, as well as vesicles of low aging propensity [21]. Besides high stability, poly-fluorinated phospholipids demonstrate polymorphic phase transitions [22], increased membrane stiffness [23] and segregations of the lipid fractions on the membrane-like lipid rafts [24]. The same effects are normally created by life on the earth with ether lipids (stability), sphingomyelin (rafts), cholesterol (stiffness) and complex lipid compositions (phase transitions). *Fluorous* lipids can represent a good alternative. Nevertheless, Effective energy storage is one particularly important function which cannot be taken over by the *fluorous* lipids.

It is experimentally verifiable that polypeptides containing perfluorinated amino acid side chains exhibit a lipophobic effect for highly fluorinated alkyl groups [25,26]. Extensively fluorinated bio-polymers such as perfluorinated amino acid-containing polymers are resistant to high temperatures and exhibit an unusual phase segregation behavior: they are simultaneously hydrophobic and lipophobic, and they preferentially interact with other fluorocarbon compounds [27]. In contrast, hydrocarbon-based folded polymeric structures such as proteins are folded into stable 3D structures by means of the hydrophobic effect,* i.e.*, by exclusion of apolar residues from the aqueous phase. In fact, proteins fold into a tight molecule with an interior hydrophobic core that is shielded from the surrounding water solvent following the general “apolar in-polar out” folding principle [28]. This should hold true for folded perfluorinated proteins as well. Perfluorinated side chains are endowed with the capacity to induce a hydrophobic collapse through their exclusion from either aqueous or organic phase as shown in Figure 1. Perfluorocarbons have been described as fluorophilic (*i.e.*, neither lipophilic nor hydrophilic) and they are not expected to interact with hydrophilic nor hydrophobic solvents [29].

It is therefore not surprising that the production of highly perfluorinated compounds triggered the attention of not only academia but also biotech companies, e.g., enzymes with such highly fluorinated structural substituents would solve two basic problems for enzyme-based bio-transformations (i) resistance towards various organic (and possible other adverse) solvents and (ii) finding new innovative solutions for problems in the production of new biomaterials, biosensors and protein therapeutics [33]. The basic rationale behind these efforts is an attempt for a rational design and construction of novel structures endowed with predetermined and unusual functions, which can be exploited in xenobiotics research to increase membrane permeability of pharmacologically active substances, thus enhancing drug activity.

### 3.3. From Fluorinated Amino Acids to Proteins and Peptides 

The simplest experimental approach to introduce fluorine into a protein structure is to perform protein biosynthesis with amino acid analogues in which single hydrogens are replaced with fluorine counterparts [34]. As fluorine is much less bulky than chlorine, it causes less steric perturbances in the protein structures. On the other hand, fluorine is the most electronegative element and subsequently C–F bonds dramatically change charge properties of molecules when compared with those of the C–H bond [35]. For example, its dipole moment is opposite to that of a C–H bond and a strong inductive effect is generated [36]. Fluorine possesses a very high ionization potential and low polarizability—therefore fluorinated molecules exhibit only weak intermolecular interactions,* i.e.*, highly fluorinated compounds are characterized by weak surface energies, and small dielectric constants and refractive indices [37]. Due to its electronegativity, fluorine-carbon-bonds are strongly polarized with the positive partial charge at the carbon atom. C–F bonds have stronger energies than the other C-halogen-bonds and also have a high bond dissociation energy. For example, fluoroalkyl chains are extremely resistant to chemical transformations. However, for amino acid building blocks with single H→F substitutions (*i.e.*, mono-fluorinated amino acids), the fluorine atom is not a sterically demanding substituent, although the C–F bond is 0.4 Å longer than the C–H bond. Therefore, monofluorinated amino acid analogues have been often used as substitutes in protein biosynthesis [38,39,40]. 

**Figure 2:**
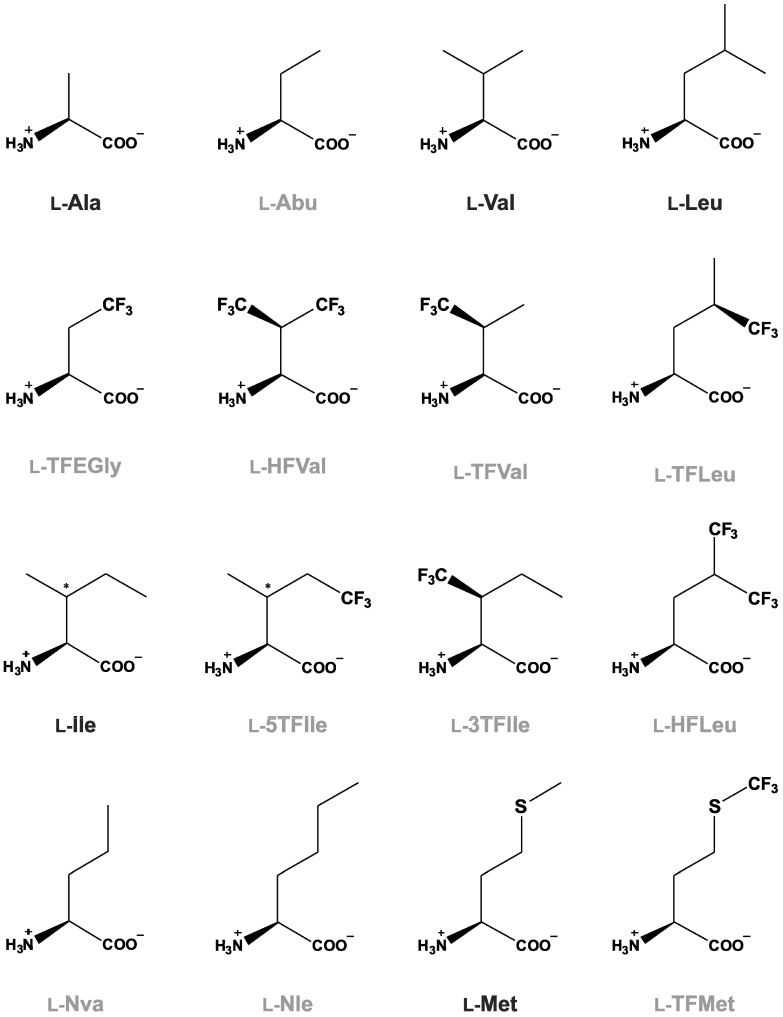
Hydrophobic canonical (Ala, Val, Leu, Ile, Met) and noncanonical amino acids (Norvaline, Nva; α-aminobutyrate, Abu; norleucine, Nle) used for building of protein cores. At least traces of Nle, Nva and Abu have been found in the interstellar medium and carbonaceous meteorites, in addition to some canonical amino acids [28]. These amino acids have perfluorinated counterparts of anthropogenic origin which have special properties. Frequently used trifluorinated amino acids are: TFMet (6,6,6-trifluoromethionine), TFLeu (5,5,5-trifluoroleucine), HFLeu—(5,5,5,5’,5’,5’-hexafluoroleucine), TFVal—(2*S*,3*R*)-4,4,4-trifluorovaline; 5TFIle [(2*S*,3*S*)-5,5,5-trifluoroisoleucine] and 3TFIle—[(2*S*,3*S*)-3,3,3-trifluoroisoleucine]. The asterisk in the structure indicates an undefined stereogenic (*i.e.*, chiral) carbon center.

The hydrophobic character of a molecule can be increased by the introduction of fluorine atoms. However, in relation to monofluorinated amino acids extensively fluorinated amino acids exhibit much more dramatic differences when compared with natural hydrocarbon counterparts. For example, they have markedly increased molecular volume, exhibit resistance to high temperatures, and show an unusual phase segregation behavior [41]. In particular, the CH_3_→CF_3_ substitution increases sterical bulk for more than 12 Å^3 ^per single replacement [42]. Figure 2 summarizes some frequently used trifluorinated amino acids in peptide and protein studies. The most prominent property of the trifluoromethyl group is its elevated hydrophobicity (it is almost twice as hydrophobic as the methyl group). However, there are still unresolved issues. For example, hydrocarbons are more polarizable than fluorocarbons (since the cohesive dispersion forces between two hydrocarbons molecules are greater than between two fluorocarbons [43]) and we still lack solid evidence for self-segregating properties of complex perfluorocarbon-containing proteins. Namely, the majority of studies on “*fluorous* proteins” was conducted using peptides mainly synthetized chemically, such as *fluorous* bio-active peptides or *fluorous* antimicrobial peptides [44]. Extensive fluorination of such small peptides preserves the shape of the related side chains, which is crucial for the correct packing of side chains within the hydrophobic core, while the increased size and hydrophobicity is well tolerated [45]. 

In contrast, the incorporation of perfluorinated amino acids into proteins in living cells is still a challenging issue for research [46]. Engineering the genetic code has recently become an important discipline in chemical biology [28] as it provides an efficient platform for the transfer of numerous synthetic molecules from the laboratory to the biochemistry of living cells, including the use of fluorinated molecules.

There are a few recent experimental reports of global incorporation of trifluorinated aliphatic amino acids into proteins of living cells [29]. First pioneering works by Rennert and Anker have demonstrated that some leucine auxotrophs of *E. coli* are capable to grow to a certain extent in the synthetic media containing trifluoroleucine (Figure 2) instead leucine [34]. The important observation was that the amino acid transport systems are not capable to distinguish between the sterically similar amino acids leucine (Leu) and trifluoroleucine (TFLeu). On the other hand, the mechanism of how they succeeded to adapt auxotrophs to grow on TFLeu remained obscure until today, since this substance is a strong inhibitor of cellular growth [47]. However, the reside-specific incorporation of noncanonical amino acids circumvents this toxicity in a quite simple and straightforward way [48]. The target gene activity must be kept silent while growing the host cells to an appropriate level. After enough “healthy” cells are produced, the synthesis of the target protein is induced. From this point on, the host cells serve only as a “factory” to produce the desired recombinant protein and their further growth is of secondary importance. An additional requirement is that the metabolic pathway that supplies the cell with the particular canonical amino acid (e.g., leucine) is switched off. This makes host cells auxotrophic,* i.e.*, dependent on the amount of the canonical amino acid present in a defined minimal medium. Furthermore, the fermentation in minimal media with growth limiting concentrations of the native amino acid as natural substrate leads to its depletion from the medium. At this point the non-canonical amino acid is added and the protein synthesis is induced, resulting in the accumulation of exclusively labeled proteins [49]. 

## 4. Life Based on Fluorine Chemistry?

Is life based on fluorine chemistry possible? It appears clear that natural scaffolds which evolved on Earth are generally not suitable for the accommodation of perfluorinated amino acids as protein building blocks. Namely, protein structural frameworks with hydrocarbon cores are shaped and optimized by billions of years of evolution on Earth and are not very suitable to accommodate a large number of fluorine atoms. Therefore, the generation of a fluorine-based biochemistry seems daunting. However, we have to make us free from our pre-conceptions, which derive solely from life on Earth, and realize that life will adapt to its environment and make use of the available resources to the fullest [50]. But, given the case that life in all its diversity on Earth never adopted fluorine as a biogenic element, its accommodation into the metabolism and physiology of organisms is indeed a formidable challenge. The question remains whether this is the case only for life on Earth, for which fluorine might have not been sufficiently available in the prebiotic evolution toward life. Or, does fluorine has properties, which makes it universally unfit for life? Certainly, fluorine is the element with the highest electronegativity, trumping oxygen, and is highly aggressive in many compounds, especially as hydrofluoric acid. And being in the sole oxidation state of −1, it cannot be used in metabolic redox-reactions in which its oxidation state changes (like for example carbon). However, cells are capable of developing enzymes for carbon-fluorine bond formation [4], indicating that metabolic engineering with organic fluorine is indeed conceivable. In addition, there is already solid evidence in the literature that the proteome-wide introduction of fluorine could be possible [51].

The arguments brought forward against fluorine as a major universal molecule of life mirrors the arguments that could be brought forward against oxygen. Oxygen has the second highest electronegativity, can be extremely aggressive (e.g., as OH radical) and is highly toxic to anaerobic organisms. Cyanobacteria were the first organism on early Earth that pumped vast amounts of oxygen into the atmosphere causing a global environmental catastrophe for the early biosphere on Earth more than 2 billion years ago [52]. At that time Earth’s biosphere consisted largely of anaerobic microbial communities. However, today, life without oxygen, especially complex life without oxygen, is unthinkable. The associated energy and energetic reactions are difficult to handle for biology, but once they can be handled those pathways are evolutionary favored and much superior to less energetic reactions. 

Given that fluorine is the 13th most common element on Earth, a terrestrial planet; it may be more often used in a biological scheme on another world. Certainly, there must be planets and moons out there, where fluorine is more common and available than on Earth. One feasible scenario on an exoplanet is that all molecular oxygen may be used up by oxidation reactions on the planet’s surface and fluorine gas is released from F-rich magma later in the history of that planetary body [9]. Or, fluorine may be much more common in solution on a planet where water is not the primary solvent, addressing its low solubility of fluoride in water. There fluorine could play a similar role oxygen plays in life on Earth, and may even lead to the evolution of animal-like organisms providing sufficient energy (or easily more) than oxygen does in aerobic metabolism. 

We might obtain a glimpse of fluorine’s potential from recent laboratory work in synthetic biology and xenobiology [53]. For example, the experimental work on microbial cell evolution might lead to cells with a proteome that consist of a novel chemical composition including alternate physiologies or novel metabolisms [54,55,56].

## 5. Summary

Life on Earth, especially complex life, uses oxygen for metabolism and a variety of other critical biochemical processes. Thus, a scenario is feasible where F, which can be used for potentially even more energetic reactions, could be used by biology. The fact that it is not used by life on Earth in any significant way does not necessarily mean that this is not possible. Most likely, the absence of the use of fluorine in Earth’s biochemistry is due to the poor availability of fluorine under early Earth conditions. The scenario might be quite different on another planetary body with a diverging planetary history, a possibility underlined by recently gained insights when producing artificial fluorinated biomolecules.
